# ‘Weighty issues’ in GP-led antenatal care: a qualitative study

**DOI:** 10.1186/s12875-019-1026-4

**Published:** 2019-10-29

**Authors:** Ruth Walker, Tammie S. T. Choi, Karyn Alexander, Danielle Mazza, Helen Truby

**Affiliations:** 1 Department of Nutrition, Dietetics and Food, School of Clinical Sciences, Monash University, Level 1, 264 Ferntree Gully Road, Notting Hill, VIC 3168 Australia; 2 Department of General Practice, School of Primary and Allied Health Care, Monash University, 246 Clayton Road, Notting Hill, VIC 3168 Australia

**Keywords:** Pregnancy, Primary care, Antenatal care, Weight, Guidelines, Barriers, Enablers, Implementation

## Abstract

**Background:**

Approximately 50% of women gain weight in excess of gestational weight gain (GWG) recommendations during pregnancy leading to adverse maternal and foetal outcomes and the perpetuation of the cycle of obesity. Antenatal care provided by a general practitioner (GP) in the primary care setting is an important model of care, particularly for women in regional areas where rates of overweight and obesity are highest. The aim of this study is to explore GPs’ perceptions and experiences of implementing GWG recommendations in GP-led antenatal care.

**Methods:**

A qualitative exploratory approach recorded GPs’ experiences and insights regarding the application of GWG recommendations in practice. Data were collected via semi-structured interviews informed by the revised Theoretical Domains Framework (TDF). Deductive thematic analysis grouped coded text into TDF domains from which main themes were generated.

**Results:**

Twenty GPs (13 female, 7 male) from metropolitan and regional Victoria, Australia participated. Codes related to at least one of 11 TDF domains. Five main themes were apparent: 1) Despite low awareness of guidelines, GWG advice is provided; 2) ‘I should do this more’; 3) Lack of everyday resources; 4) Working ‘against the odds’ at times; and 5) Optimism and reality. GPs were aware of the importance of optimal GWG however, other pregnancy-related issues are given precedence during consultations. Enablers for the implementation of GWG guidelines were practitioner-based and included GPs’ strong sense of their professional role to provide advice, and ongoing and trusting relationships with women throughout pregnancy. Barriers were mostly health system-based with limited time, remuneration, and restrictive referral pathways being limiting factors.

**Conclusions:**

There is a need to support GPs to provide GWG advice in accordance with current national guidelines. Solutions potentially lie in strategies that promote the effective dissemination and uptake of guidelines, and changes to policy and funding within the health-system so that longer GP-led antenatal care consultations are remunerated and referrals to allied health are accessible to women who require additional support to optimise GWG.

## Background

Approximately 50% of women gain weight in excess of recommendations during pregnancy [1–3]. Maternal complications associated with excessive gestational weight gain (GWG) include gestational diabetes mellitus (GDM), pre-eclampsia [4], caesarean section [2] and post-partum weight retention [5]. Adverse foetal outcomes include large for gestational age, macrosomia [2], higher levels of adiposity, and a predisposition for obesity later in life [6]. Assisting women to manage their weight during pregnancy is one element that may help break the generational cycle of obesity [7].

Recommendations for GWG vary globally however, the United States’ National Academies’ recommendations [8] are incorporated into the antenatal care guidelines of many developed countries [9–12] and frequently referred to in the literature regarding GWG [2, 13, 14]. These recommendations specify weekly and overall GWG based on pre-pregnancy weight (Table 1). Australian guidelines (2012–2017 and current) [9, 15] refer to these weight gain parameters, recommending that maternal body mass index (BMI) is calculated at the first visit and women are informed of appropriate GWG.

**Table 1:** National Academies’ recommendations for gestational weight gain in pregnancy [8]

Pre-pregnancy (BMI)	Recommended weight gain (kg)	Rate of weight gain in second and third trimesters kg/week^a^
Underweight BMI < 18.5 kg/m^2^	12.5–18.0	0.5
Healthy weight BMI 18.5–24.9 kg/m^2^	11.5–16.0	0.4
Overweight BMI 25–29.9 kg/m^2^	7.0–11.5	0.3
Obese^b^ BMI > 30 kg/m^2^	5.0–9.0	0.3

*BMI* Body Mass Index

Twins: Women of a healthy weight should gain 16.8–24.5 kg, overweight 14.1–22.7 kg, and obese 11.3–19.1 kg. There are no recommendations for triplets.

^a^Assuming 1-2 kg weight gain in 1st trimester

^b^The IOM gestational weight gain guidelines does not make any specific recommendations for women with Class II or Class III obesity

Pregnancy care provided by a family doctor or general practitioner (GP) with obstetric training in the primary care setting (GP-led antenatal care) is one of several antenatal care models available to women globally [9, 16–18]. GP-led antenatal care allows women to benefit from continuity of care with their GP from preconception to postpartum [18–20] with input from an obstetrician as required. High levels of patient satisfaction, decreased waiting times in public antenatal clinics [21] and improved access to care in rural areas [18, 20] are additional benefits. While rates of GP-led antenatal care are declining in some countries including New Zealand, Canada and the UK [18, 22], it remains an important aspect of maternity services, particularly for women living in rural communities that have experienced the closure of obstetric services [16, 20].

Women with overweight or obesity at conception are most likely to experience excessive GWG [23]. Rates of overweight and obesity tend to be higher in rural and remote areas [24, 25] where women tend to be more dependent on GP-led antenatal care due to difficulties accessing hospital-based care [9, 16, 20] adding an additional layer of complexity to case management in antenatal care in these areas. Women who choose GP-led antenatal care may have access to allied health professionals such as dietitians through hospital-based services. However, these services are often limited to pregnancies that are considered high risk (e.g. maternal BMI > 30 kg/m^2^, GDM) where hospital-based care is recommended over shared care models [26].

Health professionals agree that optimal maternal nutrition is critical for both mother and child [27–29] however, there is less accord with how to support women to achieve energy balance and prevent excessive GWG [30]. A qualitative study explored Australian GPs’ experiences and perceived role in optimising GWG [31], but no research has investigated how or whether GPs apply GWG recommendations in practice. Therefore, the aim of this study is to explore the perceptions and experiences of GPs in Australia in relation to implementing GWG recommendations in GP-led antenatal care.

## Methods

### Theoretical framework and design

A qualitative exploratory approach recorded GPs’ experiences managing GWG in GP-led antenatal care and gathered insights regarding the application of GWG recommendations in practice. Data were collected via a semi-structured interview informed by the revised Theoretical Domains Framework (TDF) [32] (Table 2), exploring 14 domains known to influence practice. The TDF integrates behaviour change theories into a single validated tool [32] widely used in implementation science to identify barriers and enablers to guideline implementation [33]. Ethics approval was obtained from Monash University Human Research Ethics Committee (Ref: CF16/789–2,016,000,384) and written consent was obtained from participants.

**Table 2:** Semi-structured interview questions mapped to the 14 domains of the Theoretical Domains Framework

Domain	Question
1. Knowledge	Can you tell me what is meant by the term *excessive maternal GWG*? Can you describe the guidelines regarding maternal GWG used at your practice?
2. Skills	What training have you completed in order to provide women with advice regarding weight management for pregnancy? How do you address the topic of weight management with women during pregnancy?
3. Social/professional role and identity	According to your guidelines regarding maternal GWG, what is your role in assisting women to manage their weight during pregnancy? What is your personal belief regarding your role in assisting women to manage their weight during pregnancy?
4. Beliefs about capabilities	Do you feel confident to discuss weight management issues with women during pregnancy? What particular abilities do you think health professionals need in order to provide weight management advice during pregnancy?
5. Optimism	When you provide advice about weight management during pregnancy, how do you expect women to respond? Do you believe that women will apply the advice that you give them about weight management during pregnancy? What do women require in order to increase their compliance to your advice?
6. Beliefs about consequences	What are the risks associated with excessive maternal GWG for women and/or infants?
7. Reinforcement	What are the incentives to take time in a consultation to offer women weight management advice during pregnancy?
8. Intentions	Do you intend to continue/make weight management advice a key aspect of your time with women in the future? Why/why not?
9. Goals	What do you consider to be the long-term benefits (for women and/or children) if you provide weight management advice during pregnancy?
10. Memory, attention and decision processes	Under what circumstances would you offer advice regarding weight management during pregnancy?
11. Environmental context and resources	What aspects of your work environment support you to offer weight management advice for women during pregnancy? What aspects of your work environment make it challenging for you to offer weight management advice for women during pregnancy? How likely are you to refer women to other health professionals as part of the care they receive? What resources do you require in order to assist women with weight management during pregnancy?
12. Social influences	Do you believe that weight management during pregnancy is important to women?
13. Emotion	To what extent do you think your emotions affect your capacity to offer advice regarding weight management for pregnancy?
14. Behavioural regulation	What procedures to you believe need to be in place in order to deliver consistent and patient-centred advice to women regarding weight management during pregnancy?

*GWG* Gestational weight gain

### Setting and participants

GP-led antenatal care affiliate databases of two large hospital networks in Victoria, Australia were used to locate GPs practising in GP-led antenatal care. Purposive sampling aimed to recruit 20 GPs with even representation of males and females from metropolitan and regional settings. Invitations to participate with research aims listed were sent to potential participants (*n* = 109) via post. Those who refused to participate did so by not replying and therefore, did not provide a reason. Participants nominated a preferred date and time for a phone interview and received a $100 gift voucher in appreciation of their time.

### Data collection and analyses

A schedule of questions mapped to the TDF were piloted with experts in maternal nutrition and general practice (Table 2). Interviews were conducted between April and August 2017 by one female research dietitian with postgraduate training in qualitative research methods (RW) and no prior relationship with participants. The researcher conducted the interviews in an office while participants were at work or at home. Interviews lasted 20–30 min, and were audio-recorded and transcribed verbatim. Participants were offered the opportunity to member-check transcripts. Deductive thematic analysis, using NVivo 9 software (QSR International Pty Ltd. 2010) grouped coded text into TDF domains, from which the main themes were generated. Coding of three randomly selected transcripts was undertaken by two researchers (RW, TC). After cross-checking for consistency all transcripts were coded. Throughout the coding process the same two researchers agreed upon how to categorise codes and identified the point when data saturation was reached before generating the main themes.

## Results

Twenty GPs (13 female) from 13 metropolitan and seven regional areas participated. The Socio-Economic Indexes for Areas (SEIFA) ranking system that considers relative socio-economic advantage or disadvantage across Australia indicated that half of the GPs practiced in the three most advantaged SEIFA deciles.

Deductive content analysis identified 53 codes relating to at least one of 11 TDF domains. Some codes applied to two or more domains. Five main themes and seven sub-themes regarding GPs’ awareness, knowledge and application of GWG guidelines in GP-led antenatal care were apparent. These themes and how they relate to the TDF are described in Table 3 and with exemplar quotes below.

**Table 3:** Themes and sub-themes mapped to the Theoretical Domains Framework. Solutions to address barriers to the implementation of GWG guidelines are mapped to the COM-B system for behaviour change (COM-B = Capability, Opportunity, Motivation - Behaviour)

Themes and sub-themes	Barrier ✗	Enabler ✓	TDF domain	Possible solution
*THEME 1:* Despite low awareness of guidelines, GWG advice is provided *Sub-theme:* Knowledge of guidelines is low *Sub-theme:* Providing advice, but only to those who need it most			Knowledge	Capability: Clear GWG guidelines Effective dissemination of guidelines
Despite low awareness of GWG guidelines, GPs provide many women with advice and support regarding weight management in pregnancy.	✗	✓		
*THEME 2:* ‘I should do this more’			Social/professional role and identity Beliefs about capabilities/ consequences	
General Practitioners considered it their professional role to support women with weight management in pregnancy.		✓		
Providing GWG advice is often deprioritised in busy consultations.	✗			
Many GPs said that they would prioritise the provision of advice about this important topic in the future.		✓		
*THEME 3:* Lack of everyday resources *Sub-theme:* Lack of time *Sub-theme:* Lack of resources *Sub-theme:* Lack of clear guidance			Skills Environmental context and resources Behavioural regulation	Capability: Clear guidelines Additional training Opportunity: Access to multidisciplinary team and/or practice nurses
Barriers to GPs providing women with weight management advice are national health policy and funding-based.	✗			
*THEME 4:* Working ‘against the odds’ at times *Sub-theme:* Meeting women where they are at *Sub-theme:* Social environment			Social influences Reinforcement	Motivation: Public health messages that promote the importance of weight management for pregnancy
Women’s motivation and perceptions of weight management in pregnancy, and the broader social environment can be barriers and/or enablers for the implementation of GWG guidelines.	✗	✓		
*THEME 5:* Optimism and reality			Optimism Environmental context and resources	Motivation: Affirmation in role
General Practitioners generally believe that primary care is an ideal setting to provide GWG advice and that women respond well to the advice they receive.		✓		
On the other hand, women’s capacity to put the advice they receive into practice is challenged by the broader physical and social environment.	✗			

### Theme 1: despite low awareness of guidelines, GWG advice is provided

Awareness of Australian and/or international GWG guidelines was generally low. Despite this, most GPs discussed GWG with women at some point during pregnancy, transposing skills and knowledge utilised when undertaking preventive care in non-pregnant populations. The following sub-themes align with the TDF domains related to knowledge [32].

#### Knowledge of guidelines is low

Four GPs made reference to a specific GWG guideline. Most knew that recommendations were based on pre-pregnancy BMI but when prompted to define excessive GWG, responses mostly fell within a range of 12-15 kg. Four GPs gave incorrect responses. Conversely, GPs demonstrated a thorough understanding of the maternal and foetal risks associated with excessive GWG.

*“I have read some [guidelines] in the past, just trying to remember where I read them … Maybe the Women’s Hospital … I have read, at least read an article on it if it wasn’t the guidelines.” (Regional GP #19)*

#### Providing advice – but only to those who need it most

Less than half of the GPs interviewed reported giving basic information regarding GWG to all women early in pregnancy*.* Most references to the provision of GWG advice were related to providing advice to women who GPs perceived required it the most (overweight or gaining too much weight) or if women ask them directly. The importance of establishing trust with patients before discussing the sensitive topic of GWG was emphasised with, *‘You can’t just launch into that [topic of GWG] straight up in the consultation. It just doesn’t work. You’ve actually got to spend a little bit of time.’* Weight management was integrated into other aspects of consultations with questions such as, *‘Have your tastes changed?’* rather than addressing GWG as a separate issue for the fear of making their patient feel uncomfortable. Four GPs reported weighing women at every visit.

### Theme 2: ‘I should do this more’

All GPs cons**idere**d the provision of GWG advice to be an important aspect of care. Factors driving this important enabler of GWG guideline implementation align with TDF domains, social/professional role and identity and beliefs about capabilities/consequences ^32^. Incentives to provide GWG advice were patient-centred, with GPs listing short and long term health-related outcomes as motivating factors.

*“And if the mum is healthy you can guarantee then that the family itself will be healthier, because she’s going to be the one who’s preparing most of the food. And then you’re setting the kids up for a good childhood and therefore a good adulthood... It’s important and it improves outcomes for mums and babies, and through that we get stronger families and stronger communities, and as a GP that’s my bread and butter business.” (Regional GP #13)*

Despite being acknowledged as an important topic, weight wasn’t always in the forefront of GPs’ minds during busy consultations. Many stated that simply participating in the interview reminded them of the importance of the topic.

*“I think I will actually, after this interview, focus on it [GWG] more than what I already do” (Regional GP #3)*

### Theme 3: lack of everyday resources

GPs described three main barriers to the implementation of GWG guidelines that aligned with TDF domains, environmental context and resources, behavioural regulation and skills [32].

#### Lack of time

Limited time in relatively short consultations seemed to work against comprehensively covering all aspects of antenatal care, let alone building and/or maintaining trust and having conversations about the sensitive topic of GWG.

*“Sometimes there are so many things to cover … They will have often brought a long list and they'll say, ‘I want my pap smear, I want this and that,’ and then in your head you're quickly prioritising things. I’ve gotta do a pap smear, we'll talk about folic acid, I've got to check their blood tests, make sure I've booked them into hospital and then plan the next ultrasound and talk about Down Syndrome … Listeriosis and mercury, and weight and alcohol and smoking and all that.” (Metropolitan GP #18)*

Two GPs reported referring women eligible for subsidised allied healthcare services (e.g. dietitian or physiotherapist) decreased their time burden. In Australia, Medicare-funded incentives give patients with a chronic disease access to subsidised allied healthcare however, pregnancy does not give women access to this funding. Five GPs suggested extending the availability of this referral pathway to pregnant women, or remuneration for longer GP-led antenatal care consultations as solutions for overcoming the issue of limited time.

#### Lack of resources

Access to practical resources such as fact sheets and brochures were considered important for supporting the advice GPs provide: ‘*The other thing that I do lack is easy resources on diet and pregnancy. You know, an easy resource that I can just give them.’* Limited access to multidisciplinary support was also highlighted as an issue.

*“Gee, what other things do they need? What other weapons? Yeah. It’s a damn good question. They probably need a hell of a lot … Someone to walk around the supermarket with them probably … I mean, maybe a few extra healthcare professionals... A good dietitian somewhere … I suppose a psychologist might be handy every now and then, or some other trick up your sleeve”. (Regional GP #6)*

Many GPs said that they did not refer because they felt they had no one to refer to, were unsure of the services in their area, or thought women received dietetic advice at the hospital. Those practising within lower socioeconomic areas were less likely to refer due to the additional cost burden for women, compared with those practising in higher socioeconomic areas who mostly referred without any reservations.

*“I’m fairly unlikely [to refer] … Here patients are very unlikely to pay for full cost of a dietitian or an exercise physiologist. In fact I haven’t come across a single patient who’s willing to do that. So therefore the access, because of financial reasons … is very difficult.” (Metropolitan GP #2)*

#### Lack of clear guidance

GPs reported little and only general training regarding nutrition or weight management, either in general medical training or when attaining their obstetric qualification. Most acknowledged that they did not have the specific skills to provide advice for more complex cases.

*“Ooh, goodness! I guess no specific training in relation to weight management … I mean, I’ve been to meetings, conferences and so forth over the years where these things have been discussed and talked about, but no specific training as such.” (Metropolitan GP #15)*

GPs suggested actions to overcome the barrier of a lack of clear guidance regarding GWG guideline implementation. These involved having clearer guidelines, easily accessible resources to provide women in order to support the advice they give, and additional training regarding weight management in pregnancy.

*“If there’s any resources developed or guidelines released, that are about weight management, that they’re either endorsed or hosted by an organisation such as the women’s hospital. Something that’s reputable and identifiable to the patients as well.” (Regional GP #9)*

*“A regular education update … Things are always changing about what dietary recommendations there are, what exercise recommendations there are … One of the things that would be good – sometimes I can’t go to the education evening because I’m a mum and I’ve got to manage my family – would be to have some of them available as webinars.” (Metropolitan GP #7)*

### Theme 4: working ‘against the odds’ at times

There were mixed findings regarding GPs’ perceptions of women’s capacity to make lifestyle changes for optimal weight gain in an ‘obesogenic’ environment where is it very easy to gain weight [34]. This theme aligned with TDF domains social influences and reinforcement [32].

#### Meeting women where they are at

When asked, ‘On a scale of one to ten, how important is GWG to women?’ responses were highly variable (ranked 3–10) with no difference between metropolitan and regional GPs. How GPs addressed the sensitive topic of GWG was influenced by whether women held misconceptions about GWG, had mental health issues, whether socio-economic factors were involved and women’s fear of GWG.

*“If you’re starting with a very sensitive patient who has got low self-esteem quite often the response can be a negative one and can be one that’s associated with tears, and can be highly emotional, the visit. So you have to be very careful what you say so you don’t turn them off you.” (Metropolitan GP #2)*

#### Social environment

GPs displayed a degree of frustration at working in a milieu where there was an abundance of energy-dense takeaway foods, busy lifestyles that squeeze out opportunity for food preparation and exercise, and a lack of cooking skills passed on from generation to generation.

*“I saw a woman last week, she came in here and dumped her Maccas on the desk. So it’s that kind of thinking and that kind of perception out in the community that it’s okay and that’s just what you do, and there’s a lack of healthy food options around here. And in some ways it’s passed down from mother to daughter that that’s just what you eat, so there’s that sort of shift that you need to try and make.” (Metropolitan GP #1)*

Despite generally being considered a protective factor for health and well-being, GPs reported that the impact of friends, family and culture is not always supportive with myths and misconceptions such as, *‘eat for two’, ‘shouldn’t exercise’, ‘I can’t have dairy’,* needing to be dispelled.

*“You have to be careful what you say in front of their husbands and partners because sometimes the husbands and partners can be quite cruel when it comes to weight gain … Also I guess cultural things. If they come from a culture where the food is very high in calories … That can be a bit difficult because that’s the norm and you’re asking them to step outside the norm.” (Metropolitan GP #2)*

### Theme 5: optimism and reality

GPs were generally optimistic about how women respond to the advice they provide however, they were less certain that women would be able to apply the advice in a broader social and physical environment that promotes weight gain. Despite GPs reporting being fairly confident to discuss GWG, they were also aware their advice may not be sufficient to support weight management during pregnancy. This theme falls under TDF theme optimism and environmental context and resources [32].

*“But in general, if I gave advice, they would follow I think at least to some degree, or do as best as they can. But some people do have difficult lifestyles and aren’t necessarily able to fit it in. So I think its variable, but in general, if they’re my patient, they tend to do pretty well with following advice.” (Metropolitan GP #14)*

GPs described how they do their best when barriers established by national health funding, *‘can outweigh … trying to help women optimise their pregnancy outcomes’.* The following quote encapsulates the tension between GPs’ perceptions of their role to provide holistic care in a clinical care setting where limited time, resources and inaccessible referral pathways are a reality.

*“There’s often eight patients waiting in the waiting room, you’re trying to sort of make everyone happy … The pressure of time makes it hard to do justice … It’s hard. I mean, that’s an issue, but that’s our problem. I guess access to professional assistance, such as nutritionists, dietitians … [that is] difficult for cost reasons or other reasons … GPs always struggle, yeah, to meet everyone’s needs. We do our best.” (Metropolitan GP #17)*

## Discussion

Eleven TDF domains are evident in this research that explored GPs’ perceptions and experiences of implementing GWG recommendations in GP-led antenatal care. GPs’ knowledge, skills, social/professional role and identity, beliefs about capabilities, beliefs about consequences, memory, attention and decision-making processes, behavioural regulation, environmental context and resources, social influences, reinforcement and optimism act as barriers or enablers for the provision of GWG advice according to GWG guidelines [32].

Low awareness of GWG guidelines was evident. This is consistent with previous research reporting that GPs tend to know guidelines for practice exist, have a general knowledge of key recommendations, but are less able to recall specific details (e.g. dose) [35, 36]. Lack of self-efficacy has also been identified as a barrier to the implementation of guidelines in general [35–37] however, this was not strongly represented by our sample in relation to the implementation of the GWG guidelines, specifically. In contrast, GPs considered supporting women with GWG as crucial to their role and were optimistic that long-term relationships assisted them to provide advice.

GPs believed their advice was generally well-received but thought the broader environment challenged women’s application of it in order to bring about lifestyle change that prevents excessive GWG. This is confirmed by women themselves who have stated that the ease of access to ‘junk food’ and social factors such as expectations from family members often make it difficult to achieve optimal GWG [38]. Additional barriers were time constraints, lack of resources and lack of training. These same barriers have been reported in general practice regarding preventive health in children [39], preconception care [40], and guideline implementation in general [35, 36].

**Fig. 1:**
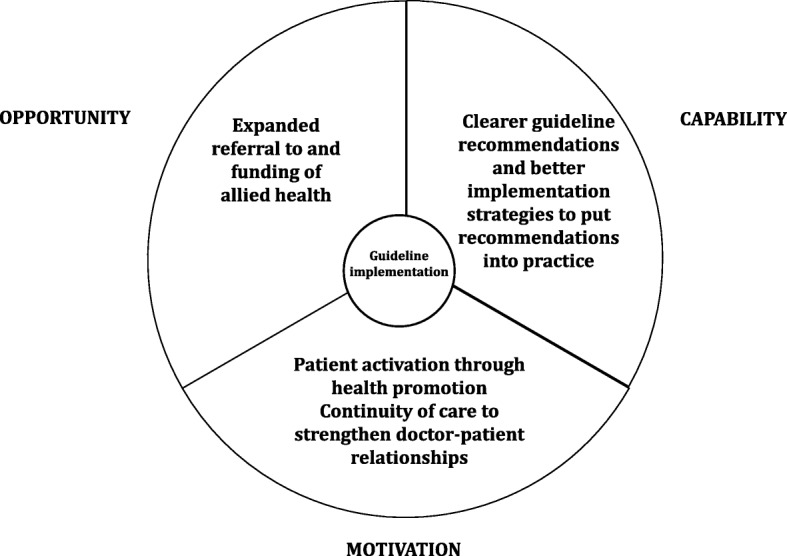
Solutions to the enhanced implementation of gestational weight gain guidelines mapped to the COM-B framework for behaviour change

The COM-B system [32] frames health professionals’ *Capability* (psychological and physical), *Opportunity* (social and physical) and *Motivation* (reflective and automatic) as key factors that drive behaviour change in healthcare [41]. The 14 domains of the TDF have been mapped to the COM-B system [32] making it a useful framework for developing interventions to overcome barriers to guideline implementation [32, 39, 42] (Table 3 and Fig. 1).

### Capability

Tentative and even incorrect definitions of excessive GWG put forward by interviewed GPs reflecting previous research conducted with obstetricians and midwives in hospital settings [43, 44]. Knowledge of guidelines impacts on practitioner capability with attitudes and behaviours being driven by knowledge [36]. Therefore, our research confirms that that some women may receive little or incorrect GWG advice in pregnancy [38, 45]. This widespread confusion regarding GWG recommendations and the provision of advice may be attributed to significant changes in GWG guidelines over the past 30 years, within the careers of many GPs [46, 47], and sub-optimal dissemination of new recommendations.

In 2000, Australia’s National Health and Medical Research Council (NHMRC) described a range of guideline dissemination strategies including mass-media campaigns, educational materials, and administrative interventions [48]. In addition to current guideline dissemination strategies, a multifaceted approach [47] involving the provision of information to professional associations and universities at conferences, developing audio-visual summaries online, and making use of social media may assist with increasing awareness. Furthermore, Kastner et al [47] identified two key domains associated with the implementability and uptake of guidelines: 1) Creation of guideline content (stakeholder involvement, evidence synthesis, considered judgement, feasibility), and 2) Effective communication of content including having multiple versions and formatting with information visualisation. Monitoring and evaluation of guideline implementation is also crucial in order to answer questions, ‘Are guidelines being followed?’ and ‘Does their implementation make a difference?’ [49].

The Australian NHMRC’s *Clinical Practice Guidelines: Pregnancy Care 2018 Edition* [9], released after the interviews for this research were conducted, place greater emphasis on weight management in pregnancy and should be seen as an opportunity to implement consistent practice across all models of antenatal care in Australia [46]. The consensus-based recommendation, *‘At every antenatal visit, offer women the opportunity to be weighed and encourage self-monitoring of weight gain’* clarifies that it is acceptable to weigh all women at every antenatal visit and to encourage women to monitor their weight in between consultations. The provision of culturally appropriate information regarding healthy eating and physical activity to promote optimal GWG is encouraged. The benefits of healthy lifestyle conversations was confirmed in a recent systematic review that found that interventions to prevent excessive GWG do not necessarily need to highly intense and costly, but should be embedded into existing models of care [50]. The new guidelines also offer some, albeit brief, advice about how this may be achieved with, *‘Adopting a respectful, positive and supportive approach and providing advice about health healthy eating and physical activity in an appropriate format may assist discussion of weight management. This should be informed by appropriate education for health professionals’* [9].

Most GPs stated that they would like more training regarding weight management in pregnancy. This could be addressed at undergraduate and post-graduate level by enhancing maternal nutrition and weight management course content and increased training opportunities in affiliated hospital networks, professional associations and Colleges [51]. Consideration should be made for those working in regional and remote areas by making material available online.

### Opportunity

Addressing the issue of limited time in consultations is crucial for enhancing the implementation of GWG guidelines. This may be possible with national health policy and funding that provides remuneration for longer consultations in antenatal care. GPs acknowledged that accessing a dietitian was not an option for some women due to cost and suggested that women would benefit from subsidised access to other members of a multidisciplinary team. This was also recommended by Van Der Plight et al [31] in an earlier study that explored GPs’ perceptions of GWG. To be eligible for subsidised access to allied health in Australia, patients must have a chronic or terminal medical condition [52]. By altering eligibility criteria to include pregnancy, women would have access to specialist advice, thus decreasing the work burden of GPs. In the United Kingdom, antenatal care guidelines recommend that women with a body mass index > 30 kg/m^2^ are referred to a dietetic service or other health professional who can offer additional lifestyle advice to promote optimal GWG [53]. Women in the UK report being satisfied with dietetic services but, at times, were disappointed with the process of referral and long waiting lists [54]. Another solution is to expand the role of practice nurses in clinics where GP-led antenatal care is provided. With appropriate training, practice nurses are well-placed to provide basic lifestyle advice that has the potential to support appropriate GWG [7], as well as information relating to other health topics that may be sensitive for women [40, 55]. In doing this, practice nurses and GPs should be cognisant of their scope of practice and refer on if required.

### Motivation

General Practitioners were motivated to provide GWG advice because of their comprehensive knowledge of risks associated with excessive GWG. This has been demonstrated previously [31]. A strong sense of responsibility and commitment was demonstrated by all GPs who consistently recounted their intention to discuss GWG with women in the future. Their high level of motivation is a strong enabling factor of guideline implementation provided that barriers to implementation are addressed. These GPs who are working against barriers to GWG guideline implementation on a daily basis suggested a range of solutions including consistent guidelines, training, and easy resources to provide women, “*without having to go from site to site and print bits and pieces from here and there”*, and longer consults. These solutions should be considered carefully and acted upon with changes to national funding, health policy, and public health messages that promote maternal nutrition and lifestyles for optimal pregnancy outcomes [3, 56].

### Strengths and limitations

There is a paucity of research regarding the role of GPs in supporting women to manage GWG in GP-led antenatal care, not to mention the role of all GPs who have clinical encounters with women throughout pregnancy for confirmation of pregnancy, pregnancy-related conditions such as *hyperemesis gravidarum*, or conditions unrelated to pregnancy. The study design was underpinned by a validated theoretical framework designed to explore guideline implementation [32]. Cross-checking between researchers occurred frequently throughout the analyses.

The sample recruited 20 participants were from Victoria, Australia and half were from the top three SEIFA deciles. Therefore, generalisability to other parts of Australia and other countries cannot be assured. Despite the sample size, the specific aim of the research that was based on validated theory and the depth of the interviews and analyses contributed to the information power [57]. Further, our results are consistent with previous research across antenatal care models confirming that the management of GWG varies in GP-led antenatal care and hospital settings [31, 43, 44]. In addition, barriers to the implementation of GWG guidelines such as lack of time, resources and training have been identified across antenatal care models [21, 43, 58] and general practice [35, 40].

## Conclusions

Overall, GPs provided women with advice that would support weight management in pregnancy despite their low awareness of actual recommendations. Awareness of GWG guidelines should be promoted with effective guideline dissemination and implementation and professional development. Behaviour modification could be further supported by national health policy and funding measures that increase women’s access to multidisciplinary support. Public health messages and policy should promote a broader environment that fosters optimal nutrition and weight management in pregnancy as crucial aspects of preventive health.

## Data Availability

All data generated or analysed during this study are available upon request.

## Abbreviations

BMI: Body Mass Index
COM-B: Capability, Opportunity, Motivation-Behaviour
GDM: Gestational diabetes mellitus
GP: General Practitioner
GWG: Gestational weight gain
SEIFA: Socio-economic Index for Areas
TDF: Theoretical Domains Framework
